# Familial Renal Glucosuria and Potential Pharmacogenetic Impact on Sodium-Glucose Cotransporter-2 Inhibitors

**DOI:** 10.34067/KID.0000000621

**Published:** 2024-10-16

**Authors:** Patrick Allaire, Jamie Fox, Terrie Kitchner, Rachel Gabor, Connie Folz, Shankar Bettadahalli, Scott Hebbring

**Affiliations:** Center for Precision Medicine Research, Marshfield Clinic Health System, Marshfield, Wisconsin

**Keywords:** pharmacokinetics, renal function, SGLT2

## Abstract

**Key Points:**

- A significant knowledge gap exists in *SLC5A2*'s role in familial renal glycosuria and sodium-glucose cotransporter-2 inhibitors' efficacy.
- Two percent of individuals in the All-of-Us cohort harbor rare genetic variants in *SLC5A2*, potentially increasing the risk of familial renal glycosuria.
- Our trial suggests differential responses to sodium-glucose cotransporter-2 inhibitors in individuals with rare *SLC5A2* alleles compared with wild types.

**Background:**

Renal glucosuria is a rare inheritable trait caused by loss-of-function variants in the gene that encodes sodium-glucose cotransporter-2 (SGLT2) (*i.e*., *SLC5A2*). The genetics of renal glucosuria is poorly understood, and even less is known on how loss-of-function variants in *SLC5A2* may affect response to SGLT2 inhibitors, a new class of medication gaining popularity to treat diabetes by artificially inducing glucosuria.

**Methods:**

We used two biobanks that link genomic with electronic health record data to study the genetics of renal glucosuria. This included 245,394 participants enrolled in the All of Us Research Program and 11,011 enrolled in Marshfield Clinic's Personalized Research Project (PMRP). Association studies in All of Us and PMRP identified ten variants that reached an experiment-wise Bonferroni threshold in either cohort, of which nine were novel. PMRP was further used as a recruitment source for a prospective SGLT2 pharmacogenetic trial. During a glucose tolerance test, the trial measured urine glucose concentrations in 15 *SLC5A2* variant–positive individuals and 15 matched wild types with and without an SGLT2 inhibitor.

**Results:**

This trial demonstrated that carriers of *SLC5A2* risk variants may be more sensitive to SGLT2 inhibitors compared with wild types (*P* = 0.075). On the basis of population data, 2% of an ethnically diverse population carried rare variants in *SLC5A2* and are at risk of renal glucosuria.

**Conclusions:**

As a result, 2% of individuals being treated with SGLT2 inhibitors may respond differently to this new class of medication compared with the general population, suggesting that a larger investigation into *SLC5A2* variants and SGLT2 inhibitors is needed.

## Visual Abstract

**Figure:**
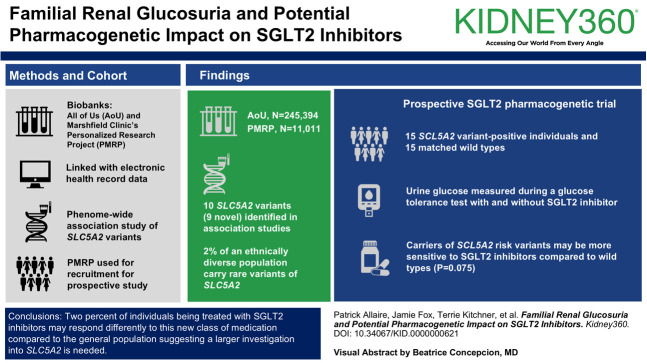

## Introduction

Over 462 million individuals worldwide are affected with type 2 diabetes. By 2030, it is projected that the disease will affect over 7% of the population,^1^ with highest rates in Western societies where high caloric diets and sedentary lifestyles are common. Type 2 diabetes comes with high morbidity and mortality. Numerous pharmacologic agents have been developed to treat type 2 diabetes. This includes a recent class of medication called sodium-glucose cotransporter-2 (SGLT2) inhibitors, a drug class that has also been repurposed to treat obesity and elevated systolic and diastolic BP.^2^

SGLT2 is predominantly expressed on the luminal surface of epithelial cells in the proximal tubule of the kidney. SGLT2 functions as a monomeric symporter, simultaneously transporting two sodium ions and one glucose molecule from the urine into the cell.^3^ Under normal conditions, 97% of glucose that enters the renal lumen is reabsorbed by SGLT2. The remaining 3% is attributed to sodium-glucose cotransporter 1.^4^

Current SGLT2 inhibitors are predominantly glucose analogs with bulky sidechains that work *via* competitive inhibition to prevent glucose reabsorption resulting in glucosuria, the excretion of glucose through the urine. Although SGLT2 inhibitors were initially approved for treatment of type 2 diabetes with additional off-label uses (*e.g*., obesity), they have since been US Food and Drug Administration approved to improve cardiovascular and renal outcomes when treating individuals with chronic heart failure and kidney disease (with or without diabetes mellitus).^5^ In the United States, there are currently six US Food and Drug Administration–approved SGLT2 inhibitors: bexagliflozin, canagliflozin, dapagliflozin, empagliflozin, ertugliflozin, and sotagliflozin, with additional SGLT2 inhibitors used in other countries. SGLT2 inhibitors generally show appealing pharmacokinetic parameters with high oral bioavailability, long elimination *t*_1/2_, limited renal excretion, no active metabolites, few drug–drug interactions, and low accumulation index.^6^ SGLT2 inhibitors are associated with a variety of side effects, the most clinically concerning of which include urinary tract infections, electrolyte imbalances, diabetic ketoacidosis, increased risk of bone fracture, lower limb amputation, and possibly bladder cancer.^7^ Given the importance of SGLT2 inhibitors when treating patients with diabetes and cardiovascular and/or renal disease, it is clinically relevant to understand factors that may affect variation in response for this class of medications.

**Figure 1:**
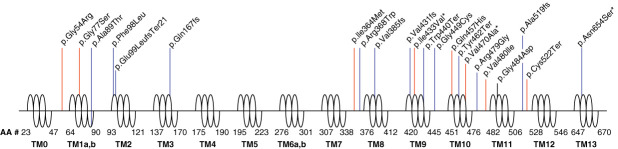
**SGLT2 protein with TM domains defined by AA#.**^31^ Graphed are all known protein altering pathogenic/likely pathogenic variants (blue)^9,10^ and novel variants associated with renal or all glucosuria (red). *Variants studied in the prospective trial. AA#, amino acid number; SGLT2, sodium-glucose cotransporter-2; TM, transmembrane.

SGLT2 is encoded by the gene *SLC5A2.* Rare pathogenic variants in *SLC5A2* have been characterized to cause an inheritable form of glucosuria referred to as renal glucosuria. Renal glucosuria is generally a benign asymptomatic trait, but some may be at risk for extreme thirst, excess urination, and dehydration. Similar to those on a SGLT2 inhibitor, individuals with renal glucosuria are also at risk for urinary tract infections because of excess glucose in the urine. Renal glucosuria has been characterized as a Mendelian trait reported to be both recessively^8–10^ and dominantly^10–12^ inherited. Unless symptomatic, most patients with renal glucosuria may be diagnosed only through an incidental finding. To date, 27 pathogenic or likely pathogenic (P/LP) variants in *SLC5A2* have been cataloged as possible risk factors for renal glucosuria according to ClinVar.^10^ Most documented variants (66%) have been missense variants and are distributed toward the N- and C-terminus of SGLT2 (Figure 1).

Owing to the usually benign nature of renal glucosuria, the trait is likely under diagnosed. The genetic etiology of this disorder remains incomplete. We therefore hypothesized that additional variants in *SLC5A2* may cause renal glucosuria. We further hypothesized that individuals with SLC5A2 variants may respond differently to SGLT2 inhibitors. To better characterize the genetics of renal glucosuria and its pharmacogenetic impact on antidiabetic medications designed to induce glucosuria, we evaluated *SLC5A2* genetics in two large biobanks linked to electronic health record (EHR) data. We further studied how these variants affect SLGT2 inhibition *via* a prospective drug trial.

## Methods

### Cohort Descriptions

#### All of Us Research Program

All of Us (AoU) is a resource created by the National Institutes of Health with the intent to recruit 1,000,000 or more participants across the United States from diverse backgrounds.^13^ Participants can consent to share EHR data and/or DNA for research. Although AoU participants are recruited across the United States by many organizations with regulatory oversight from a central Institutional Review Board (IRB), de-identified research data are available to the scientific community and IRB exempt.

**Table 1:** Demographics of different subsets of AoU and Personalized Research Project biobanks

Demographic Variable	AoU		PMRP		
			Cohort	Prospective Trial	
	AoU Participants with WGS (*N*=245,388)	AoU Participants with WGS+EHR (*N*=182,497)	PMRP (*N*=11,011)	Wild Type (*n*=15)	Variant Positive (*n*=15)
**Ethnicity, *No.* (%)**					
Admix-American	45,034 (18)	31,679 (17)	0 (0)	0 (0)	0 (0)
African	56,911 (23)	38,306 (21)	77 (0.7)^a^	0 (0)	0 (0)
East Asian	5706 (2.3)	3443 (1.9)	0 (0)	0 (0)	0 (0)
European	133,578 (54)	106,216 (58)	10,934 (99.3)	15 (100)	15 (100)
Middle Eastern	942 (0.38)	706 (0.39)	0 (0)	0 (0)	0 (0)
South Asian	3217 (1.3)	2147 (1.2)	0 (0)	0 (0)	0 (0)
**Sex at birth, *No.* (%)**					
Male	94,756 (39)	67,477 (37)	4333 (39)	1 (6.7)	1 (6.7)
Female	145,563 (59)	111,330 (61)	6678 (61)	14 (93)	14 (93)
Unknown	5069 (2.1)	3690 (2.0)	0 (0)	0 (0)	0 (0)
Mean years of age	54.5±SD17.0	55.5±SD16.9	70.88±SD15.5	59.8±SD11.7	59.7±SD12.4
Mean years of EHR data		9.64±SD8.4	36.40±SD7.9		
Mean BMI				31.9±SD5.5	32.0±SD6.0

AoU, All of Us; BMI, body mass index; EHR, electronic health record; PMRP, Personalized Research Project; WGS, whole genome sequencing.

a Include all non-European ancestries.

We used AoU's Controlled Tier Dataset version C2022Q4R9, released on April 20, 2023. This included 245,394 adults with whole genome sequence data, including a subcohort of 182,497 individuals with EHR data. The demographic data of the cohort and subcohort are reported in Table 1. Genetic data were restricted to the exome call set variants (exon regions of the Gencode v42 basic transcripts +15 bases on either side of each exon) at the *SLC5A2* locus. Variant annotation was performed using the provided AoU variant annotation table and limited to the main *SLC5A2* Ensembl transcript ENST00000330498.4 (protein ENSP00000327943.3). Association testing was performed using logistic regression on the basis of International Classification of Diseases (ICD) coding and urine glucose laboratory measurements (Table 2). Sex, age at last visit, length of EHR, 16 genetic principal components, and disease comorbidities were included as covariates (Supplemental Table 2). For laboratory measurements, we used all available semiquantitative dipstick test results defined by the Observational Medical Outcomes Partnership concept identifications,^14^ excluding those for specific conditions, such as pregnancy. Values between 0 and 100 mg/dl were excluded because of the assay's imprecision and limit of detection. Participants with at least two laboratory values ≥100 mg/dl were defined as cases for glucosuria (*n*=6272); all others were defined as controls (*n*=148,018). Because of low counts in genotypes and some phenotypes, we applied conservative Firth's bias-reduced logistic regression (FIRTH) and Fisher exact tests. All *P* values are two-tailed.

**Table 2:** Electronic health record–defined phenotypes

Phenotype Definition	ICD9	ICD10	SNOMED	OMOP Concept ID
**Primary case**				
All glucosuria	271.4, 791.5	E74.8, E74.81, E74.810, E74.818,E74.819, E74.89, R81	n/a	n/a
Renal glucosuria	271.4	E74.818	n/a	n/a
Urinalysis	n/a	n/a	n/a	3024629, 3009261, 3039896, 2212166, 3020650, 3030260, 3020399
**Covariates**				
Diabetes mellitus	250.XX code series	E10.XXX and E11.XXX code series	73211009	
Renal disease	403.XX, 404.XX, 585.XX, and 586.XX code series	I12.XX, I13.XX, I15.XX, N17.XX,N18.XX, N19.XX code series	n/a	

Because primary phenotypes (*i.e*., renal and all glucosuria) were rare, only one code of any code (rule of one) was required to define a case. For common covariates (*i.e*., diabetes mellitus and renal disease), at least two or more of any relevant code (rule of two) was required to define a case. All others were controls. ICD9, International Classification of Diseases, 9th Revision; ICD-10, International Classification of Diseases, 10th Revision; ID, Identification; OMOP, observational medical outcomes partnership; SNOMED, systematized nomenclature of medicine.

### Phenome-Wide Association Study of SLC5A2 Variants

The phenome-wide association study (PheWAS) was conducted using logistic regression with the PheWAS R package.^15^ We included all CLINVAR-defined P/LP variants and our significant findings (*n*=2633 carriers). A carrier was defined as an individual with any of these variants, whereas wild-type individuals carried none. Covariates included sex, age at last visit, length of EHR, and 16 genetic principal components. Disease status was determined by ICD codes mapped to phecodes (version 1.2; https://phewascatalog.org/phewas/#phe12).^16^ Individuals coded with the same phecode on two or more distinct dates were considered cases; those without a phecode were controls. Individuals with only a single phecode were excluded. Only phenotypes with at least 20 cases were considered for the PheWAS.

#### Personalized Medicine Research Program

Personalized Research Project (PMRP) is a biobank of 20,000 adult Marshfield Clinic patients who have consented to share EHR and genomic data.^17–19^ Over 98% of PMRP are White non-Hispanic patients, with 77% claiming German ancestry (Table 1). Of the 20,000, 11,011 participants were genotyped on a custom-modified HumanCoreExome array by Illumina, Inc. for the study of age-related macular degeneration.^20^ This population represented PMRP participants over at least 5 years of EHR data (mean=36.4 years of EHR data). The single nucleotide polymorphism array data captured eight variants predicted to alter SGLT2 protein sequence. Five were monomorphic, whereas three were polymorphic (p.[Val470Ala], rs139661242; p.[Ile433Val], rs150546732; and p.[Asn654Ser], rs61742739).

### Prospective Trial

PMRP was the recruitment source for the prospective trial. The prospective trial was approved by Marshfield Clinic's IRB, and all participants provided written informed consent. On the basis of association testing results and available genotype data, three renal glucosuria risk variants were selected for the study (p.[Val470Ala], p.[Ile433Val], and p.[Asn654Ser]). Fifteen healthy variant-positive carriers and 15 healthy age-, sex-, and BMI-matched wild types (Table 1) were recruited into the prospective trial. Exclusion criteria included those older than 80 years or those diagnosed with renal disease, diabetes, or glucosuria. To ensure participants were healthy and eligible, a board-certified endocrinologist manually evaluated EHR data, and participants were surveyed before enrollment and start of trial activities. Because of rarity, individuals heterozygous for p.(Val470Ala) and p.(Ile433Val) were oversampled.

On day 1 of the trial, all fasting participants were subjected to a standard clinical glucose tolerance test using GluCola containing 75 g of glucose (Fisher Scientific). Urine samples were collected at 15 and 120 minutes after glucose bolus. Blood was also collected before glucose bolus to confirm normal kidney function (*via* circulating serum creatinine levels) and blood glucose levels at 30, 60, and 120 minutes to monitor serum glucose response (Supplemental Figure 1 and Supplemental Table 4). During day 2, fasting participants repeated day 1 procedures but with a single dose of the SGLT2 inhibitor (empagliflozin, 10 mg, Lilly-Boehringer Ingelheim) administrated 15 minutes before glucose bolus. All experiments were conducted under Clinical Laboratory Improvement Amendments–College of American Pathologists standards at Marshfield Clinic Laboratories. Urine glucose was measured *via* the hexokinase glucose-6-phosphate dehydrogenase method performed on the Beckman Coulter Chemistry Analyzer. The lower limit of detection was 10 mg/dl. Blood creatinine levels were measured using a modified Jaffe kinetic fixed assay, and blood glucose levels were measured with a hexokinase glucose-6-phosphate dehydrogenase–based assay. Both assays were performed on the Beckman Coulter chemistry analyzer.

All protocols adhered to the Declaration of Helsinki.

## Results

### AoU

To study the genetics of renal glucosuria, we evaluated all *SLC5A2* coding variants sequenced in 245,394 participants enrolled in AoU (Table 1). A total of 1344 unique variants were identified. Of these, 820 variants had the potential to alter SGLT2 protein coding sequence. The protein altering variants were observed across all ethnicities, but were more common in African American individuals, as expected.^21^ Only two protein altering variants had allele frequencies above 1% (Supplemental Table 2).

Fifty-nine of the 820 variants had ClinVar^10^ annotation, including 11 defined as P/LP with no conflicting interpretations (Figure 1 and Supplemental Table 2). These 11 variants were observed in 243 of 245,388 individuals with genomic data. Of 182,497 AoU participants with available EHR and genomic data (Table 1), including 27 diagnosed with renal glucosuria, 182 were carriers of P/LP variants. Renal glucosuria was associated with P/LP genotype (Fisher exact *P* = 0.027; OR, 38.6; 95% confidence interval [CI], 0.936 to 237) demonstrating ability to identify expected associations. Given its benign nature, we speculated that renal glucosuria could be undiagnosed or misdiagnosed. As such, we evaluated anyone diagnosed with all forms of glucosuria (*n*=671). Associations between all glucosuria and P/LP alleles became more significant (Fishers exact *P* = 6.49E-05; odds ratio [OR], 9.31; 95% CI, 3.36 to 20.8).

To identify potentially novel *SLC5A2* variants associated with renal glucosuria, we screened all protein altering variants provided there were at least two individuals in the cohort with the variant (*n*=357 variants). Given the rarity of the alleles, rarity of renal glucosuria, and to control for nongenetic variables that may contribute to glucosuria (*i.e*., diabetes and renal disease), we applied a FIRTH logistic regression (Supplemental Table 2). In total, six variants were associated with renal glucosuria (FIRTH *P* < 0.05) (Table 3 and Supplemental Table 3). Of the six, two passed a conservative Bonferroni threshold (FIRTH *P* < 1.40E-4), including a frameshift variant previously reported to be associated with renal glucosuria (p.[Glu99LeufsTer21], rs770214617)^10^ and a novel splice variant (chr16:31487314 [C,T]). None of the 27 individuals diagnosed with renal glucosuria carried any more than one protein-altering allele of any kind, including copy number variants.

**Table 3:** Variants associated with renal, all glucosuria, and urinalysis in AoU and PMRP biobanks.

Annotations					AoU			PMRP
					Renal Glucosuria	All Glucosuria	Urinalysis	Renal Glucosuria
					*n*=27	*n*=671	*n*=6272	*n*=10
Position (hg38) or Amino Acid Change^a^	Consequence	RSid	ClinVar^10^	ESM1b LLR score^21^	*P* Value	*P* Value	*P* Value	*P* Value
chr16:31,487,314 (T,C)	splice_region_variant			NA	2.93E-05^b^	2.68E-04	0.00908	NA
p.(Glu99LeufsTer21)	frameshift_variant	rs770214617	LP	NA	3.14E-05^b^	4.04E-04	0.0104	NA
p.(Met382Thr)	missense_variant	rs141627694	VUS	−11.08 (LP)	1.77E-04	0.0235	0.623	NA
p.(Cys522Ter)	stop_gained	rs199795513		NA	0.00129	1.58E-05^b^	6.21E-04	NA
p.(Val470Ala)	missense_variant	rs139661242	VUS	−8.45 (LP)	0.003	0.109	0.00775	2.46E-05
p.(Pro602Leu)	missense_variant	rs61746400		−6.06 (LB)	0.0441	0.498	0.822	NA
p.(Ile433Val)	missense_variant	rs150546732	LB, VUS	−2.94 (LB)	0.0504	0.0555	0.0639	0.00223
p.(Gly77Ser)	missense_variant	rs368277683		−9.42 (LP)	NA	4.87E-05^b^	0.00147	NA
p.(Val480Ile)	missense_variant	rs761264242		−5.84 (LB)	NA	5.02E-05^b^	0.00688	NA
p.(Gln457His)	missense_variant	rs764491154		−13.27 (LP)	NA	8.29E-05^b^	NA	NA
p.(Ile364Met)	missense_variant			−8.95 (LP)	NA	9.59E-05^b^	NA	NA
p.(Gly54Arg)	missense_variant	rs373741171		−12.82 (LP)	NA	1.25E-04^b^	NA	NA
p.(Ala417Thr)	missense_variant	rs1346754449		−8.44 (LP)	NA	1.60E-04	NA	NA
p.(Gln167ArgfsTer20)	frameshift_variant	rs267607067	P	NA	NA	3.55E-04	0.0035	NA
p.(Arg130Cys)	missense_variant	rs760532652		−9.84 (LP)	NA	4.13E-04	0.00853	NA
chr16:31,488,878 (A,C)	splice_acceptor_variant			NA	NA	4.54E-04	NA	NA
p.(Gly332Ala)	missense_variant			−13.13 (LP)	NA	8.48E-04	NA	NA
p.(Arg368Trp)	missense_variant	rs148410166	LP/P	−9.03 (LP)	NA	8.55E-04	NA	NA
chr16:31,488,878 (A,C)	splice_region_variant	rs752072314		NA	NA	0.00132	0.0519	NA
p.(Leu606Phe)	missense_variant	rs200862764		−5.13 (LB)	NA	0.0014	NA	NA
p.(Phe535del)	inframe_deletion	rs772594068		NA	NA	0.00146	0.2259	NA
p.(Pro23Thr)	missense_variant	rs1382300816		−8.15 (LP)	NA	0.00162	NA	NA
p.(Ile76Val)	missense_variant	rs750542045		−4.2 (LB)	NA	0.00195	0.166	NA
c.1452C>G(p.(Gly484=))	splice_region_variant	rs1481942949		NA	NA	0.00262	NA	NA
p.(Leu57Pro)	missense_variant	rs1477114969		−14.54 (LP)	NA	0.00367	3.31E-04	NA
p.(Leu147PhefsTer40)	frameshift_variant	rs2082491230		NA	NA	0.00427	NA	NA
p.(Asp273Val)	missense_variant	rs748324537		−12.31 (LP)	NA	0.00485	0.55	NA
p.(Gln168Arg)	missense_variant	rs1294044448		−6.31 (LB)	NA	0.00644	0.218	NA
p.(Arg60Cys)	missense_variant	rs201586410		−11.49 (LP)	NA	0.00856	3.33E-04	NA
p.(Ala180Thr)	missense_variant	rs748172348		−5.25 (LB)	NA	0.0104	NA	NA
p.(Ala169ValfsTer18)	frameshift_variant	rs768095044		NA	NA	0.0182	0.558	NA
p.(Leu473_Val477del)	inframe_deletion	rs774846949		NA	NA	0.0243	0.8028	NA
chr16:31,488,773 (G,A)	splice_donor_variant	rs554372141	P	NA	NA	0.0246	3.61E-05^b^	NA
p.(Leu265Pro)	missense_variant			−12.95 (LP)	NA	0.0266	0.00958	NA
p.(Asn654Ser)	missense_variant	rs61742739	B/VUS/LP	−8.38 (LP)	NA	0.0273	0.0155	0.0877

AoU, All of Us; B, benign; ESM1b LLR, evolutionarily stable motif 1b log-likelihood ratio; LB, likely benign; LP, likely pathogenic; NA, not available; P, pathogenic; PMRP, Personalized Research Project; rsID, reference SNP cluster identification number; VUS, variant of unknown significance.

a Amino Acid Change based on Ensembl transcript ENST00000330498.4 (protein ENSP00000327943.3).

b *P* values are statistically significant given multiple comparison testing.

When evaluating the 671 individuals diagnosed with all forms of glucosuria, 26 variants were associated (FIRTH *P* < 0.05), including four previously linked to renal glucosuria^10^ (Table 3 and Supplemental Table 3). Of the 26, six novel variants passed a conservative Bonferroni threshold (FIRTH *P* < 1.40E-4), including one that was nominally associated with renal glucosuria (FIRTH *P* < 0.05) (Figure 1). Similar to renal glucosuria, nearly everyone (99.4%) diagnosed with all forms of glucosuria carried no more than one protein-altering variant.

Together with renal and all glucosuria, 35 variants were associated (FIRTH *P* < 0.05), including 23 missense variants. Of the missense variants, most (70%) were considered LP on the basis of annotations publicly available and derived *via* a deep protein language model (Table 3).^22^ Of the eight variants significantly associated with renal and all glucosuria (FIRTH *P* < 1.40E-4), seven were novel variants. These novel variants congregated toward the N- and C- terminus of SGLT2, like previously reported P/LP variants in ClinVar (Figure 1).^10^ To complement ICD coding data, we leverage urine glucose measurements available in AoU. All variants significantly associated with ICD coding were also associated with laboratory values (*P* < 0.05). The top two variants associated with urinalysis were two known pathogenic alleles (Table 3 and Supplemental Table 4).

Given that loss-of-function *SLC5A2* variants cause glucosuria and pharmacologic induction of glucosuria *via* SGLT2 inhibition is used to treat diabetes, we hypothesized that *SLC5A2* variants will reduce the risk of diabetes. Moreover, SGLT2 inhibitors have been reported to be protective for a variety of other traits, such as heart failure, CKD, cardiovascular disease, weight management, and BP control.^23–27^ With this in mind, we consolidated the statistically significant and known P/LP variants together and conducted a PheWAS using curated PheCodes.^16^ Although not statistically significant, given the multiple comparison testing, variants were associated with an increased risk of glucosuria (*P* = 5.09E-4; OR, 2.80; 95% CI, 1.57 to 4.99). They were also protective for type 2 diabetes (*P* = 0.019; OR, 0.88; 95% CI, 0.79 to 0.98). The strongest association that reached statistical significance after considering multiple comparison testing (*P* < 3.00E-5) was an association with infection/inflammation of internal prosthetic device, implant, and graft (*P* = 2.83E-6; OR, 2.10; 95% CI 1.54 to 2.87) (Supplemental Figure 2 and Supplemental Table 5). No *P* value inflation in the PheWAS analysis was observed (lambda=0.962, Supplemental Figure 3).

### PMRP

We further evaluated renal glucosuria in an independent biobank called PMRP at Marshfield Clinic. Of the 11,011 PMRP participants with exome SNP array data, ten were diagnosed with renal glucosuria. The exome SNP array genotyped eight *SLC5A2* protein-altering variants; five were monomorphic. Of the three polymorphic missense variants, two were significantly associated with renal glucosuria and passed an experiment-wise Bonferroni threshold (*P* < 0.00625) (p.[Val470Ala], FIRTH *P* = 2.46E-5; p.[Ile433Val], FIRTH *P* = 0.00223), whereas a third variant marginally approached significance in PMRP (p.[Asn654Ser], FIRTH *P* = 0.0877) (Figure 1 and Table 3). When compared with AoU, p.(Val470Ala) was associated, whereas p.(Ile433Val) was nearly associated, with both renal and all glucosuria. Furthermore, p.(Asn654Ser) was associated with all glucosuria in AoU with some evidence of being pathogenic,^9^ but with conflicting interpretations in ClinVar.^10^ Given the totality of association results across both cohorts and previous reports, these three variants are likely risk factors of renal glucosuria. When considering these three variants together with previously reported P/LP variants (Supplemental Table 2) and others that passed a conservative Bonferroni threshold in AoU (Table 3), 2.0% of individuals in the multiethnic AoU population were carriers and at risk of renal glucosuria.

### Prospective Study

**Figure 2:**
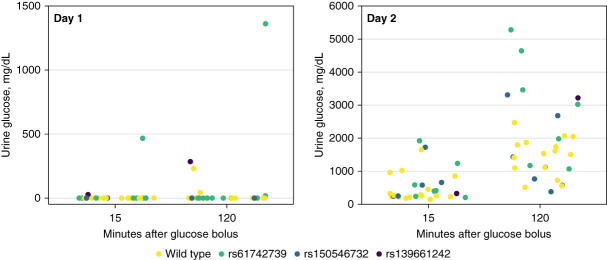
**Urine glucose measurements at 15 and 120 minutes after glucose bolus.** Included are results for day 1 (no empagliflozin) and day 2 (with empagliflozin). Colored data points indicate variant type.

Although our association results suggest that there are numerous variants that contribute to renal glucosuria, our understanding of the genetic effects of *SLC5A2* remain incomplete. Even less is known regarding the genetic effects for individuals taking SGLT2 inhibitors designed to induce glucosuria. This is important given the prevalence of the variants and how often this class of medication is prescribed. Based on EHR and genomic data from AoU, there were 4675 participants with a history of taking an SGLT2 inhibitor. Like the larger population estimates, 1.8% (*n*=85) carried a risk allele. From a different perspective, although numbers are small (<20), 7.5% of those diagnosed with glucosuria and carried a risk allele had a history of taking an SGLT2 inhibitor. We piloted a small 2-day prospective study (Supplemental Figure 1 and Supplemental Table 4). Fifteen healthy PMRP participants heterozygous for one of three risk variants (p.[Val470Ala], *n*=1; p.[Ile433Val], *n*=5; p.[Asn654Ser], *n*=9) were recruited, along with 15 healthy age-, sex-, and BMI-matched wild types (Table 1). None of the participants had been diagnosed with renal glucosuria. Fifteen minutes after glucose bolus, two variant carriers (p.[Val470Ala] and p.[Asn654Ser]) had detectable glucose in the urine (≥10 mg/dl), whereas none of the wild types expressed glucosuria (Fisher exact *P* = 0.483). After 120 minutes, one additional variant-positive participant (p.[Asn654Ser]) and two wild types had detectable glucose in the urine (Fisher exact *P* = 1.0). Although these associations were not significant, a variant carrier for p.(Asn654Ser) was a strong outlier at 15 (467 mg/dl) and 120 (1361 mg/dl) minutes suggesting that this person may have renal glucosuria (Figure 2).

**Figure 3:**
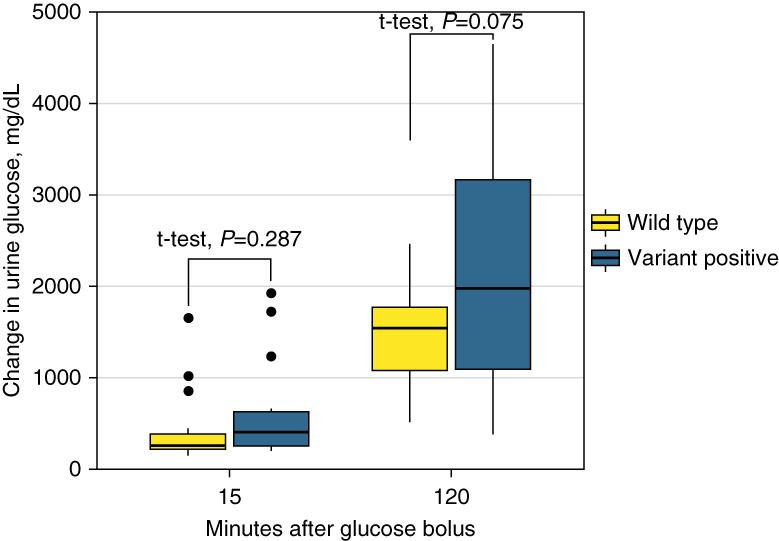
Change in urine glucose concentration between day 1 (no empagliflozin) and day 2 (with empagliflozin).

During day 2, all participants repeated the glucose challenge test while taking a single dose of SGLT2 inhibitor empagliflozin (10 mg). At 15 minutes, all participants developed glucosuria, with increasing effect at 120 minutes (Figure 2). When comparing change in urine glucose measurements between day 1 and 2, variant-positive participants at 15 minutes had a higher change in urine glucose (mean change=639 mg/dl) compared with wild types (mean change=445 mg/dl), but this difference was not significant (*t* test *P* = 0.287). At 120 minutes, the mean difference increased and approached significance (variant positive: 2166 mg/dl versus wild type: 1457 mg/dl, *t* test *P* = 0.075) (Figure 3).

## Discussion

The genetics of renal glucosuria has previously been reported as a Mendelian trait that is either recessively or dominantly inherited.^8,9,11,12^ This study suggests that the genetics of renal glucosuria is more complex with an unlikely 1:1 relationship between genotype and phenotype. All individuals diagnosed with renal glucosuria carried only one protein altering allele, including known P/LP variants in *SLC5A2* and any one of the nine novel variants this study identified (Table 2). These associations were informed *via* ICD and glucosuria laboratory values. This observation likely rules out a recessive model as the predominant inheritance pattern of renal glucosuria.

Data also suggest that a dominant model is not a common inheritance pattern for renal glucosuria. If this trait was dominantly inherited, variant carrier and disease frequencies should be similar. This was not observed. In AoU, 2.0% of the population carried a risk allele (0.1% were known P/LP alleles), whereas 0.01% were diagnosed with renal glucosuria. Incomplete EHR data or individuals being undiagnosed or misdiagnosed with the asymptomatic trait could influence the discrepancy in frequencies. For example, 0.09% of PMRP participants were diagnosed with renal glucosuria and had four times more longitudinal EHR data than AoU (Table 1). Conversely, our prospective trial demonstrated that most individuals who carried a risk allele did not express the trait even when subjected to a standard clinical glucose challenge test (Figure 2). Because of these findings, renal glucosuria could be influenced by variants with additive effects or those with significant incomplete penetrance, although we cannot rule out the possibility that renal glucosuria would have expressed itself at higher glucose concentrations or with other untested variants. Family studies may help to clarify the penetrance of SGLT2 variants. We also cannot preclude natural compensatory mechanisms that could mitigate SGLT2 dysfunction, including sodium-glucose cotransporter 1,^4^ that was not examined in this study. For reference, the single 75-g glucose bolus given to participants represented 150% of an average person's daily recommended added sugars on the basis of a 2000-calorie diet.^28^ It also equates to approximately 1.5 times more sugar than a single serving of cola (16 fluid ounce, 52 g of sugar).

*SLC5A2* variants may affect health beyond glucosuria. PheWAS analyses of risk alleles were collectively protective against type 2 diabetes. It should be noted this association did not exceed statistical significance given the multiple comparison testing, but a protective effect may be expected given that pharmacological inhibition of SGLT2 is used to treat type 2 diabetes. Our PheWAS results did not identify any other protective effects that would coincide with SGLT2 inhibition, such as heart failure, CKD, cardiovascular disease, weight management, and BP control.^23–27^ Conversely, we did pick up a statistically significant association with infection/inflammation of internal prosthetic device, implant, and graft. The cause of this association is uncertain. One explanation could be that infections in one part of the body can increase the risk of infections in other parts *via* the blood stream. For example, it has been reported that up to 13% of all knee replacement infections may be caused by oral bacteria.^29^ A similar etiology could be hypothesized in those with chronic urinary tract infections caused by genetic forms of glucosuria.

Although studying the genetics of renal glucosuria has some value when understanding a generally benign trait associated with some clinical outcomes, the greatest value may be related to better treatments for those taking an SGLT2 inhibitor designed to artificially induce glucosuria. Protein-altering variants identified in this study are presumed loss-of-function alleles; however, it is recognized the actual impact on protein function remains largely unknown. Follow-up cell line and animal model studies could help evaluate biological function of each variant. Although it is not possible to evaluate in our population because of variant frequency, individuals who are homozygotes for presumed loss-of-function variants may have limited therapeutic benefit when treated with a SGLT2 inhibitor. In other words, it is not possible to pharmacologically inhibit SGLT2 when it is already genetically knocked out. Conversely, treating individuals who maintain one functional allele (*i.e*., heterozygotes) may see glucosuria exacerbation because the drug may be more potent when there is less wild-type protein. Physiologically, increase glucose loss in the urine may result in higher risk for volume depletion and urinary tract infections. Although not statistically significant using a two-tailed test, our small prospective study supports that heterozygotes may have more sensitivity to SGLT2 inhibitors than homozygous wild types (Figure 3). If this biological model can be validated, individuals heterozygous for loss-of-function *SLC5A2* variants may receive a reduced dosage of SGLT2 to achieve the same therapeutic effect and possibly avoid unnecessary side effects.

Although this study associated many rare variants across *SLC5A2* in multiple ancestries to evaluate a rare and likely underdiagnosed phenotype that can affect statistical interpretations, the prospective drug trial focused on the biological impact of one SGLT2 inhibitor (empagliflozin) and tested only in White non-Hispanic population. Moreover, PMRP, like AoU, is approximately 60% woman because of more women being willing to participate in research than men. This sex bias was further exacerbated in the drug trial that could not be addressed with oversampling because of rarity of variants. Finally, the prospective drug study evaluated three variants based on available genomic data. Although these three variants had some level of associations across both cohorts, they did not necessarily have the largest effect sizes according to AoU results. To fully evaluate the genetics of renal glucosuria and its pharmacogenetic effect on SGLT2 inhibitors, future studies should subject heterozygotes and homozygotes over a wide spectrum of variants in a diverse population to different SLGT2 inhibitors and with varying glucose concentrations. This will be a challenging study design given the rarity of alleles, possible varying magnitude of effects for different alleles, and inherent challenges enrolling patients into clinical trials, especially healthy individuals. Conversely, such a study may be possible in large biobanks where participants with preexisting genomic data can be invited to participate in future research, similar to AoU's ancillary study Nutrition for Precision Health.^30^ If our results are confirmed, future studies will also be needed to empirically evaluate these variants' impact on drug efficacy and risk of adverse drug events for SGLT2 inhibitors. These are necessary if best practices are ever developed to titrate SGLT2 inhibitors on the basis of *SLC5A2* genotype.

In conclusion, this is the first study to comprehensively evaluate the genetics of renal glucosuria in a diverse population and its potential impact on pharmacogenetics. Although the mode of inheritance for renal glucosuria remains complex, 2.0% of an ethnically diverse population carry rare variants in *SLC5A2* that are likely risk factors of this understudied trait. Given the growing disease burden of type 2 diabetes and other common diseases affecting hundreds of millions worldwide who may be treated with SGLT2 inhibitors, this study may eventually lead to more tailored treatments for a class of medication with growing popularity.

## Supplementary Material

## Data Availability

All data are included in the manuscript and/or supporting information.

## Supplemental Material

Supplemental Figure 1. Illustration of clinical workflow of the prospective pharmacogenetic trial. Participants were required to fast for 8 hours and a fasting blood glucose sample was collected before the start of the study. Participants underwent two separate oral glucose tolerance test (OGTT), on 2 separate days, in the absence (A) and the presence (B) of the SGLT2 inhibitor, empagliflozin. The study began on administration of a glucose solution. Urine samples were collected at 15 and 120 minutes. Blood samples were collected at 30, 60, and 120 minutes.

Supplemental Figure 2. Manhatan plot of PheWAS of SLC5A2 variants.

Supplemental Figure 3. QQ plot of PheWAS of SLC5A2 p-value results.

Supplemental Table 1. Statistical models for FIRTH regression.

Supplemental Table 2. Frequencies and annotation for all coding variants genotyped in All of Us. Based on human reference genome GRCh38/hg38.

Supplemental Table 3. Summary statistic of association study. NA when no case-carrier exist to calculate association statistics.

Supplemental Table 4. Participant laboratory data from prospective study.

Supplemental Table 5. PheWAS of composite SLC5A2 score.
