# Supplementary material for: Familial Renal Glucosuria and Potential Pharmacogenetic Impact on Sodium-Glucose Cotransporter-2 Inhibitors
Source: Kidney360. 2024 Oct 16;6(4):521–30. doi: 10.34067/KID.0000000621 (PMC12045503; doi:10.34067/KID.0000000621)
Supplement: Supplementary file 1 [file kidney360-6-521-s001.pdf]

ASN
American Society of Nephrology
JASN
Journal of the American Society of Nephrology
KIDNEY360
Accessing Our World From Every Angle
CJASN
Clinical Journal of the American Society of Nephrology

ASN Journal Disclosure Form

As per ASN journal policy, I have disclosed any financial relationship or commitment held by myself and/or my spouse/partner in the past 36 months as included below. I have listed my Current Employer below to indicate there is a relationship requiring disclosure. If no relationship exists, my Current Employer is not listed.

P. Allaire reports the following:
Employer: Marshfield Clinic Health System

I understand that the information above will be published within the journal article, if accepted, and that failure to comply and/or to accurately and completely report the potential financial conflicts of interest could lead to the following: 1) Prior to publication, article rejection, or 2) Post-publication, sanctions ranging from, but not limited to, issuing a correction, reporting the inaccurate information to the authors' institution, banning authors from submitting work to ASN journals for varying lengths of time, and/or retraction of the published work.

Name: Patrick Allaire
Manuscript ID: K360-2024-000047R1
Manuscript Title: amilial renal glycosuria and potential pharmacogenetic impact on SGLT2 inhibitors Renal glycosuria and pharmacogenetic of SGLT2 inhibitors
Date of Completion: April 4, 2024
Disclosure Updated Date: April 4, 2024

ASN American Society of Nephrology
JASN Journal of the American Society of Nephrology
KIDNEY360 Accessing Our World From Every Angle
CJASN Clinical Journal of the American Society of Nephrology

ASN Journal Disclosure Form

As per ASN journal policy, I have disclosed any financial relationships or commitments I have held in the past 36 months as included below. I have listed my Current Employer below to indicate there is a relationship requiring disclosure. If no relationship exists, my Current Employer is not listed.

S. Bettadahalli reports the following:
Employer: Marshfield Clinic Health System

I understand that the information above will be published within the journal article, if accepted, and that failure to comply and/or to accurately and completely report the potential financial conflicts of interest could lead to the following: 1) Prior to publication, article rejection, or 2) Post-publication, sanctions ranging from, but not limited to, issuing a correction, reporting the inaccurate information to the authors' institution, banning authors from submitting work to ASN journals for varying lengths of time, and/or retraction of the published work.

Name: Shankar Bettadahalli
Manuscript ID: K360-2024-000047R2
Manuscript Title: Familial renal glycosuria and potential pharmacogenetic impact on SGLT2 inhibitors Renal glycosuria and pharmacogenetic of SGLT2 inhibitors
Date of Completion: August 30, 2024
Disclosure Updated Date: August 30, 2024

ASN American Society of Nephrology JASN Journal of the American Society of Nephrology KIDNEY360 Accessing Our World From Every Angle CJASN Clinical Journal of the American Society of Nephrology

ASN Journal Disclosure Form

As per ASN journal policy, I have disclosed any financial relationships or commitments I have held in the past 36 months as included below. I have listed my Current Employer below to indicate there is a relationship requiring disclosure. If no relationship exists, my Current Employer is not listed.

C. Folz reports the following:
Employer: Marshfield Clinic Health System

I understand that the information above will be published within the journal article, if accepted, and that failure to comply and/or to accurately and completely report the potential financial conflicts of interest could lead to the following: 1) Prior to publication, article rejection, or 2) Post-publication, sanctions ranging from, but not limited to, issuing a correction, reporting the inaccurate information to the authors' institution, banning authors from submitting work to ASN journals for varying lengths of time, and/or retraction of the published work.

Name: Connie M. Folz
Manuscript ID: K360-2024-000047R2
Manuscript Title: Familial renal glycosuria and potential pharmacogenetic impact on SGLT2 inhibitors Renal glycosuria and pharmacogenetic of SGLT2 inhibitors
Date of Completion: August 30, 2024
Disclosure Updated Date: August 30, 2024

ASN Journal Disclosure Form

As per ASN journal policy, I have disclosed any financial relationships or commitments I have held in the past 36 months as included below. I have listed my Current Employer below to indicate there is a relationship requiring disclosure. If no relationship exists, my Current Employer is not listed.

J. Fox reports the following:
Employer: PreventionGenetics part of Exact Sciences; and Ownership Interest: PreventionGenetics part of Exact Sciences.

I understand that the information above will be published within the journal article, if accepted, and that failure to comply and/or to accurately and completely report the potential financial conflicts of interest could lead to the following: 1) Prior to publication, article rejection, or 2) Post-publication, sanctions ranging from, but not limited to, issuing a correction, reporting the inaccurate information to the authors' institution, banning authors from submitting work to ASN journals for varying lengths of time, and/or retraction of the published work.

Name: Jamie C Fox
Manuscript ID: K360-2024-000047R2
Manuscript Title: Familial renal glycosuria and potential pharmacogenetic impact on SGLT2 inhibitors Renal glycosuria and pharmacogenetic of SGLT2 inhibitors
Date of Completion: October 3, 2024
Disclosure Updated Date: October 3, 2024

ASN
American Society of Nephrology
JASN
Journal of the American Society of Nephrology
KIDNEY360
Accessing Our World From Every Angle
CJASN
Clinical Journal of the American Society of Nephrology

ASN Journal Disclosure Form

As per ASN journal policy, I have disclosed any financial relationships or commitments I have held in the past 36 months as included below. I have listed my Current Employer below to indicate there is a relationship requiring disclosure. If no relationship exists, my Current Employer is not listed.

R. Gabor reports the following:
Employer: Marshfield Clinic Research Institute

I understand that the information above will be published within the journal article, if accepted, and that failure to comply and/or to accurately and completely report the potential financial conflicts of interest could lead to the following: 1) Prior to publication, article rejection, or 2) Post-publication, sanctions ranging from, but not limited to, issuing a correction, reporting the inaccurate information to the authors' institution, banning authors from submitting work to ASN journals for varying lengths of time, and/or retraction of the published work.

Name: Rachel Gabor
Manuscript ID: K360-2024-000047R2
Manuscript Title: Familial renal glycosuria and potential pharmacogenetic impact on SGLT2 inhibitors Renal glycosuria and pharmacogenetic of SGLT2 inhibitors
Date of Completion: September 26, 2024
Disclosure Updated Date: September 26, 2024

ASN American Society of Nephrology | JASN Journal of the American Society of Nephrology | KIDNEY360 Accessing Our World From Every Angle | CJASN Clinical Journal of the American Society of Nephrology

ASN Journal Disclosure Form

As per ASN journal policy, I have disclosed any financial relationship or commitment held by myself and/or my spouse/partner in the past 36 months as included below. I have listed my Current Employer below to indicate there is a relationship requiring disclosure. If no relationship exists, my Current Employer is not listed.

S. Hebbring reports the following:
Employer: Marshfield Clinic Research Institute

I understand that the information above will be published within the journal article, if accepted, and that failure to comply and/or to accurately and completely report the potential financial conflicts of interest could lead to the following: 1) Prior to publication, article rejection, or 2) Post-publication, sanctions ranging from, but not limited to, issuing a correction, reporting the inaccurate information to the authors' institution, banning authors from submitting work to ASN journals for varying lengths of time, and/or retraction of the published work.

Name: Scott J. Hebbring
Manuscript ID: K360-2024-000047R1
Manuscript Title: Familial renal glycosuria and potential pharmacogenetic impact on SGLT2 inhibitors Renal glycosuria and pharmacogenetic of SGLT2 inhibitors
Date of Completion: April 3, 2024
Disclosure Updated Date: April 3, 2024

ASN American Society of Nephrology JASN Journal of the American Society of Nephrology KIDNEY360 Accessing Our World From Every Angle CJASN Clinical Journal of the American Society of Nephrology

ASN Journal Disclosure Form

As per ASN journal policy, I have disclosed any financial relationships or commitments I have held in the past 36 months as included below. I have listed my Current Employer below to indicate there is a relationship requiring disclosure. If no relationship exists, my Current Employer is not listed.

T. Kitchner reports the following:
Employer: Marshfield Clinic research Institute

I understand that the information above will be published within the journal article, if accepted, and that failure to comply and/or to accurately and completely report the potential financial conflicts of interest could lead to the following: 1) Prior to publication, article rejection, or 2) Post-publication, sanctions ranging from, but not limited to, issuing a correction, reporting the inaccurate information to the authors' institution, banning authors from submitting work to ASN journals for varying lengths of time, and/or retraction of the published work.

Name: Terrie Kitchner
Manuscript ID: K360-2024-000047R2
Manuscript Title: Familial renal glycosuria and potential pharmacogenetic impact on SGLT2 inhibitors Renal glycosuria and pharmacogenetic of SGLT2 inhibitors
Date of Completion: October 8, 2024
Disclosure Updated Date: October 8, 2024
