# Supplementary figures and images for: Familial Renal Glucosuria and Potential Pharmacogenetic Impact on Sodium-Glucose Cotransporter-2 Inhibitors
Source: Kidney360. 2024 Oct 16;6(4):521–30. doi: 10.34067/KID.0000000621 (PMC12045503; doi:10.34067/KID.0000000621)

Supplemental Figure 2. Manhattan plot of PheWAS of SLC5A2 variants

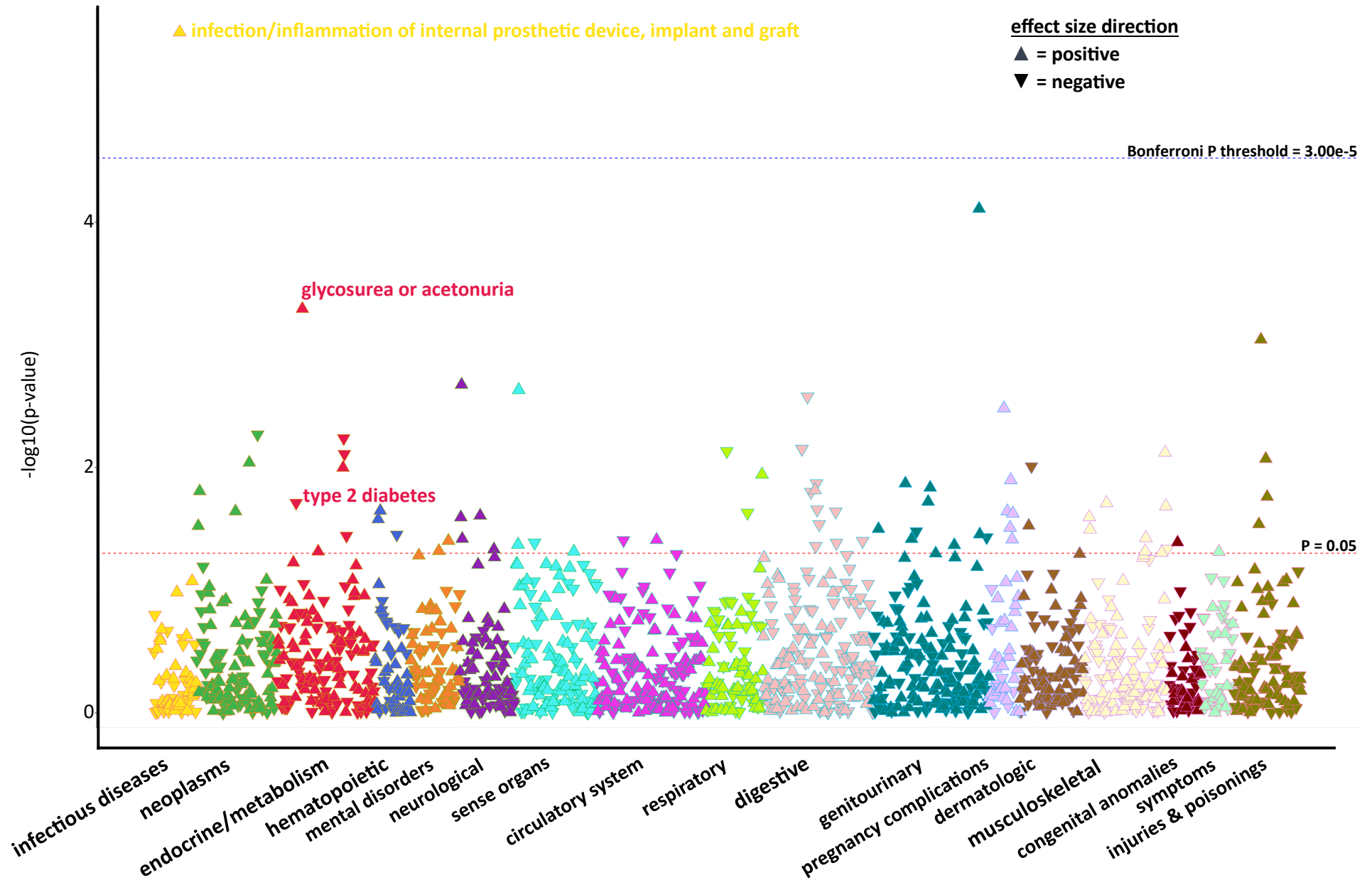

Supplement: Supplementary file 2 [file kidney360-6-521-s002.pdf]

Supplemental Figure 3 – QQ plot of PheWAS of SLC5A2 p-values results

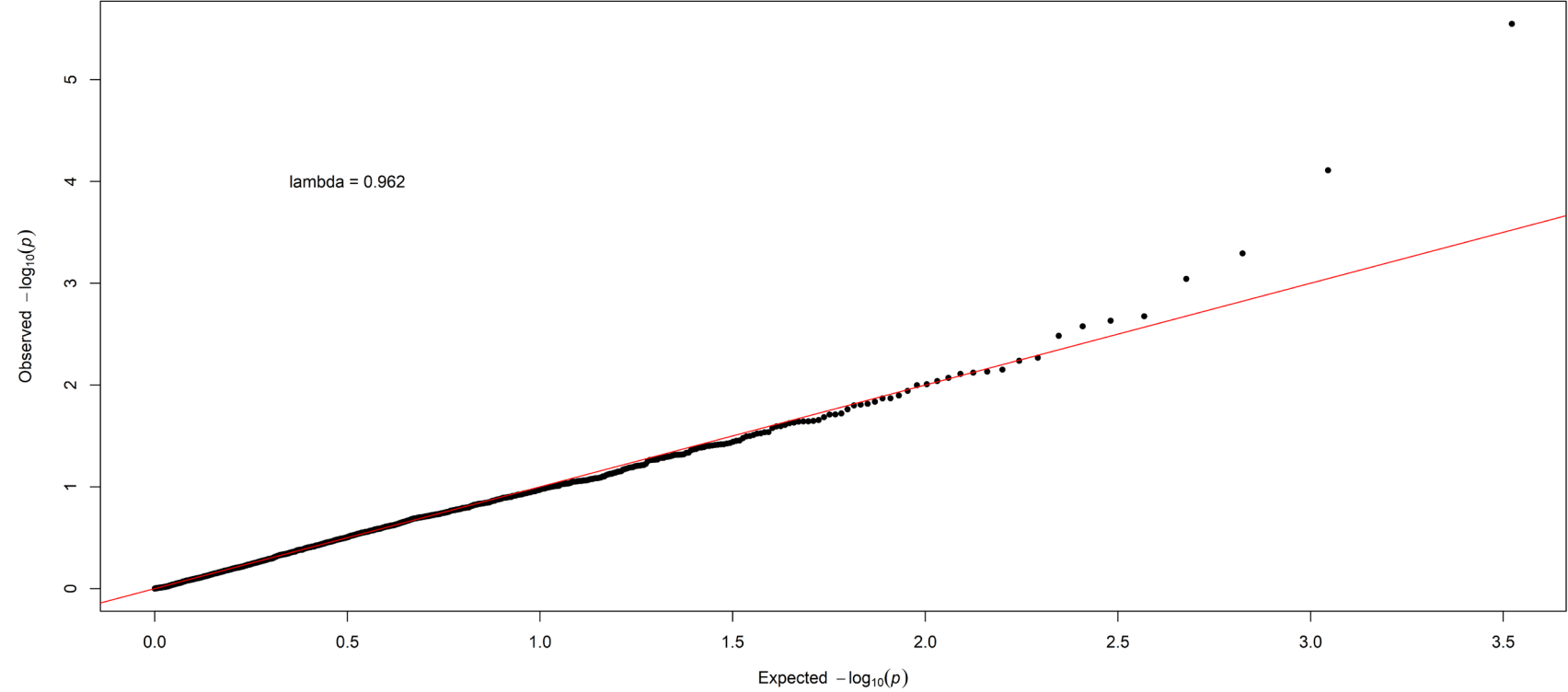

Supplement: Supplementary file 3 [file kidney360-6-521-s003.pdf]
