# Supplementary material for: Familial Renal Glucosuria and Potential Pharmacogenetic Impact on Sodium-Glucose Cotransporter-2 Inhibitors
Source: Kidney360. 2024 Oct 16;6(4):521–30. doi: 10.34067/KID.0000000621 (PMC12045503; doi:10.34067/KID.0000000621)
Supplement: Supplementary file 4 [file kidney360-6-521-s004.pdf]

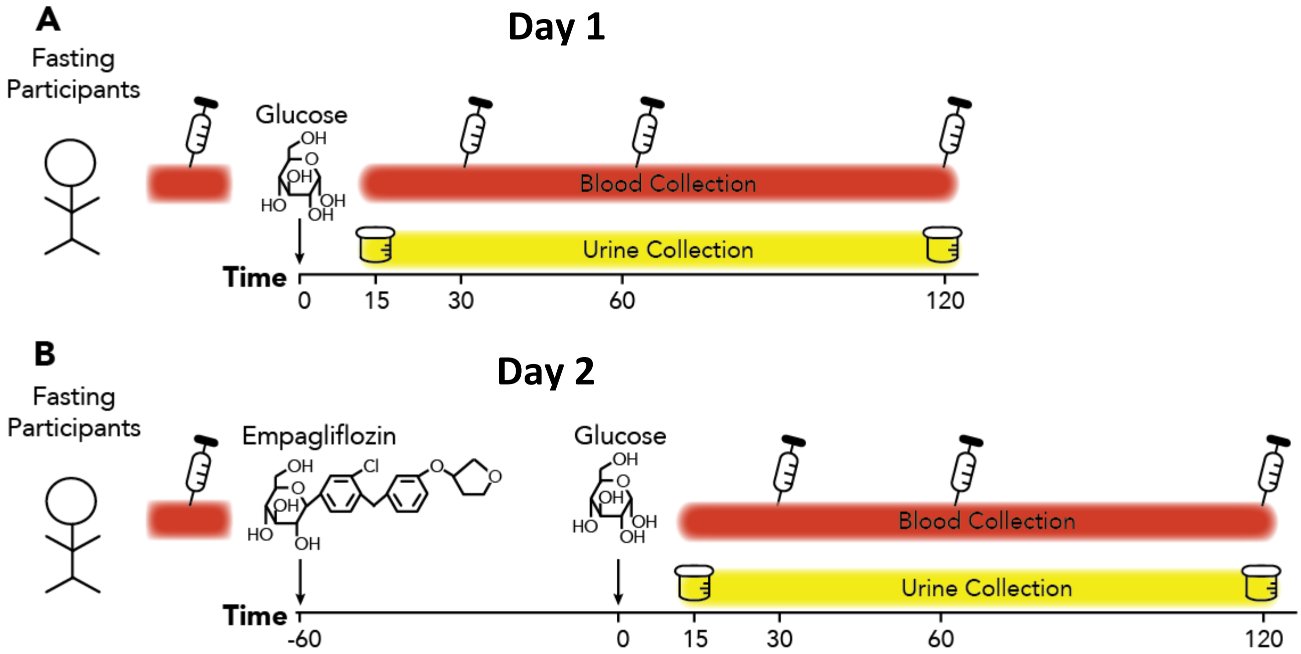

Supplemental Figure 1: Illustration of clinical workflow of the prospective pharmacogenetic trial. Participants will be required to fast for 8 hours and a fasting blood glucose sample will be collected prior to the start of the study. The participant will undergo two separate oral glucose tolerance test (OGTT), on two separate days, in the absence (A) and the presence (B) of the SGLT2 inhibitor, Empagliflozin. The study will begin upon administration of a glucose solution. Urine samples will be collected at 15 and 120 min. Blood samples will be collected at 30, 60, and 120 min.
